# Graft versus host disease: New insights into A_2A_ receptor agonist therapy

**DOI:** 10.1016/j.csbj.2014.12.003

**Published:** 2014-12-14

**Authors:** Karlie R. Jones, Elizabeth M. Kang

**Affiliations:** Laboratory of Host Defenses, National Institute of Allergy and Infectious Diseases, National Institutes of Health, Bethesda, MD 20892, USA

**Keywords:** Graft versus host disease, Adenosine, Adenosine A_2A_ receptor agonist, Regulatory T cell, FoxP3, Inflammation

## Abstract

Allogeneic transplantation can cure many disorders, including sickle cell disease, chronic granulomatous disease (CGD), severe combined immunodeficiency (SCID) and many types of cancers. However, there are several associated risks that can result in severe immunological reactions and, in some cases, death. Much of this morbidity is related to graft versus host disease (GVHD) [1]. GVHD is an immune mediated reaction in which donor T cells recognize the host as antigenically foreign, causing donor T cells to expand and attack host tissues. The current method of treating recent transplant patients with immunosuppressants to prevent this reaction has met with only partial success, emphasizing a need for new methods of GVHD treatment and prevention. Recently, a novel strategy has emerged targeting adenosine A_2A_ receptors (A_2A_R) through the use of adenosine agonists. These agonists have been shown in vitro to increase the TGFβ-induced generation of FoxP3^+^ regulatory T cells (T_regs_) and in vivo to improve weight gain and mortality as well as inhibit the release of pro-inflammatory cytokines in GVHD murine models [2,3]. Positive results involving A_2A_R agonists in vitro and in vivo are promising, suggesting that A_2A_R agonists should be a part of the management of clinical GvHD.

## 1 Introduction

Graft versus host disease (GVHD) is an immune driven disorder where donor T cells react and proliferate in response to host antigens. This leads to an immune reaction that can affect several target organs including liver, skin, and the gastrointestinal tract. GVHD remains one of the leading causes of morbidity and mortality associated with allogeneic transplantation in patients and stands as a significant barrier to the broader use of hematopoietic stem cell transplantation (HSCT) [4]. The current method of GVHD prevention is the use of immunosuppressants including calcineurin inhibitors, to reduce the donor T cell reaction to host tissues. Unfortunately, this treatment is only effective approximately 50% of the time and can have several dangerous side effects [5].

More recently, an alternate pathway of interest has been the use of regulatory T cells (T_regs_) to increase immune tolerance and prevent GVHD. Infusion of regulatory T cells has been tried in a number of studies with varying success and the commonly used immunosuppressant Sirolimus (also known as rapamycin) appears to predominantly work through T_reg_ generation [6]. Augmenting T_reg_ cells can also be achieved through the use of adenosine agonists, specifically A_2a_ receptor (A_2a_R) agonists. It has been shown that the development and increase in immunosuppressive capabilities of T_regs_ are under adenosinergic regulation and require adenosine receptor activation for their proper function [7]. Further, A_2a_R agonists have been shown to reduce inflammation and increase immune tolerance in several disease models, including ischemia, colitis and sickle cell disease [8–11]. This method has also been shown to have positive effects on T_reg_ numbers and function in GVHD animal models and to increase overall survival after allogeneic bone marrow transplantation [2,3]. Based on these promising results, it can be concluded that A_2a_R agonists via the induction of T_reg_ formation has a therapeutic potential for the treatment of GVHD.

## 2 Graft versus host disease

GVHD has an overall incidence of 35–50% [4] for all transplants. This incidence is based on patient risk factors, including donor cell source, age of the patient and pre-transplant conditioning. Acute GVHD progresses rapidly, typically occurring within 100 days of HSCT. Symptoms include dermatitis, cutaneous blisters, abdominal pain, persistent nausea and vomiting [12]. At the molecular level, GVHD is divided into three stages of progression [13]. The first stage consists of inflammation damage to host tissues by pre-transplant chemo or radiotherapy conditioning. This inflammation causes the release of pro-inflammatory cytokines such as IL-1, IL-6, IL-12, and TNF-α. These cytokines activate host antigen presenting cells (APCs), which triggers the second phase of acute GVHD. During this phase, donor T cells recognize and react to host APCs, causing an overall increase in the number of donor-derived effector T cells, both CD4^+^ and CD8^+^ cytotoxic cells [4]. Donor CD4^+^ T cells are further skewed towards a Th1 polarization, causing production of the IL-2 and IFN-γ cytokines. These Th1 cytokines encourage additional cytotoxic T-cell proliferation and cytokine release, initiating the tissue damage generally associated with acute GVHD [4,13]. Finally, during the third phase, further tissue damage occurs due to continuing expression of pro-inflammatory cytokines and donor cytotoxic T cells. The majority of this damage is a result of the Fas/FasL and perforin/granzyme B pathways as well as an effect of the activation of additional macrophages, neutrophils, eosinophils, B cells and T cells. The resulting induction of apoptosis within host tissues leads to the visible tissue damage typical of acute GVHD. Prevention or arrest of this self-destructive cycle is essential in the development of treatments for GVHD.

Currently, the standard treatment for the prevention of GVHD is the use of immunosuppressive therapy. Cyclosporin (CsA), and other calcineurin inhibitors like Tacrolimus, are commonly used to prevent GVHD as they inhibit T-cell proliferation and IL-2 production. Unfortunately, calcineurin inhibitors also have significant side effects, including hypertension, nephrotoxicity, neurotoxicity, and liver cholestasis [1]. Corticosteroids have also been used to suppress the immune response and reduce inflammation, but they are non-specific and can lead to opportunistic infections [14]. Thus there is a need for a more targeted and effective treatment/preventative for GVHD.

## 3 Adenosine, Foxp3 and the induction of immune tolerance

An important aspect of the human immune system, in addition to protecting the host from pathogenic invasions, is the ability to suppress immunological reactions to self-antigens and prevent excessive immune responses that may become damaging to the host. This activity is primarily mediated by regulatory T cells (T_regs_) that specialize in immunosuppression [15]. T_regs_ are characterized as CD4^+^CD25^+^ expressing high levels of the transcription factor forkhead box p3 (FoxP3). There are two types of T_regs_, those that arise in the thymus (natural) and those in which FoxP3 expression is activated in the periphery (induced) [16]. The importance of T_regs_ can clearly be seen in mouse models lacking CD4^+^CD25^+^ T_regs_ and in mice lacking FoxP3 expression. These mice develop fatal autoimmune diseases, allergies, and immunopathology characteristic of chronic tissue inflammation [17–20]. These changes have also been observed in human patients lacking proper expression of the FoxP3 gene (known as immunodeficiency, polyendocrinopathy, X-linked, or IPEX) and results in immune dysregulation leading to several autoimmune disorders [21–23]. Because disruption of T_reg_ function alone is enough to cause dysregulation of self-tolerance, it can be concluded that T_regs_ are essential for proper function of immunosuppression.

The purine nucleoside adenosine is released by many cells in the body and is produced mainly through the breakdown of ATP. Extracellular adenosine binds to a family of G protein-coupled receptors expressed on the surface of cells known as adenosine receptors. These fall into four subtypes including A_1_R, A_2A_R, A_2B_R and A_3_R [24]. Of particular interest is the A_2A_R subtype, as it is highly expressed in the spleen, thymus and brain [25]. This subtype is responsible for modulation of the inflammatory response and has activity on nearly all inflammatory cells. A_2A_ knockout mice have demonstrated the importance of A_2A_ receptors: the induction of acute hepatitis in A_2A_ deficient mice results in severe inflammatory tissue damage and reinforces the non-redundant significance of the A_2A_ receptor pathway in inflammation [26].

Recent data has shown that T_reg_ activation and function falls under the control of extracellular adenosine. T_regs_ have been shown to express high levels of CD39 and CD73, which are responsible for the conversion of ATP to AMP (mediated by CD39) and ultimately adenosine (mediated by CD73) [27,28]. It also appears that this adenosine, through the adenosine A_2A_ receptor pathway, inhibits the activation of effector T cells and increases the overall number of T_regs_, creating an immunosuppressive effect [7,29]. Because T_reg_ suppressive function is highly regulated by adenosine and the A_2A_ receptor pathway, it is a natural target for pharmaceutical development. However, as adenosine non-specifically binds to all adenosine receptors, both pro- and anti-inflammatory, and has a short half-life, its therapeutic value has been somewhat limited [30]. More recently, very specific adenosine A_2A_R agonists have been developed and tested in many areas, including cancer, autoimmune diseases and inflammatory disorders [31]. Use of A_2A_R agonists in the treatment of a mouse model of acute endotoxemia and sepsis improved mouse survival and decreased the levels of live bacteria in blood [32]. A_2A_ receptor activation was also shown to reduce ischemia reperfusion injury in mice [33]. Because of the success of A_2A_R agonists in other autoimmune and inflammatory diseases, interest has increased in the use of A_2A_R agonists in the treatment of GVHD.

## 4 Adenosine, adenosine receptors and their role in GVHD

GVHD is characterized by an immune response of donor T cells to host tissues, causing tissue inflammation and damage. The majority of the tissue damage in GVHD is due to the activity of alloreactive T cells, and current data suggests that the inhibition of these cells can limit morbidity associated with this disorder. To date, there are several treatments available for T cell reduction, but many of these have severe side effects, such as an increased incident of infection. In many diseases, extended administration of adenosine or adenosine analogs has reduced inflammation in tissues including the liver, skin and gastrointestinal tract with no observed side effects [34,35]. Prolonged oral treatment of a mouse model of colitis with the A_2A_R agonist ATL313 attenuated the disease with no major negative outcomes [9]. In addition, the A_2A_R agonist ATL146e has been shown to be effective for the treatment of inflammation and renal injury associated with diabetic nephropathy [36]. As a result of these findings, adenosine has become of special interest in the search for more effective treatments for GVHD.

The inflammatory reaction in GVHD is followed by the release of ATP from dying cells. This ATP is converted to AMP and further to adenosine by CD39 and CD73 respectively. This pathway has been shown to be essential and exhibit a non-redundant role in inflammatory modulation [26]. Increases in inflammatory tissue damage cause an increase in adenosine release, which creates a negative feedback loop via the A_2A_ receptor. Released adenosine binds to A_2A_ receptors on the cell surface of alloreactive donor T cells and inhibits T cell activation by host antigen presenting cells (APCs) [31]. As was previously reported, adenosine is also essential in inducing the development of T_regs_ and increasing immune tolerance [7,29]. Taking these results together, the A_2A_ receptor can mediate both innate and adaptive immune responses, reducing alloreactive donor T cells and increasing overall T_reg_ levels. It has been shown that the number of T_regs_ found in the peripheral blood and other affected tissues of patients are decreased in GVHD [37]. It is possible that this reduction in T_regs_ and increase in alloreactive donor T cells observed in GVHD could be reversed by the administration of A_2A_ receptor agonists.

## 5 A_2a_R agonists and their application in GVHD

Those treatments that limit alloreactive T cell activation and/or migration to affected tissues in GVHD have been the most promising [38,39]. Agents that elevate cAMP have also been known to prevent mortality and morbidity in murine models of GVHD [40]. The A_2A_R agonist ATL146e, which activates the cAMP elevating G-coupled A_2A_ receptor, has been shown to protect a murine model of GVHD from disease progression [3]. Acute GVHD was induced by transferring MHCI mismatched parental T lymphocytes into F1 recipients, causing extensive donor cell engraftment, release of pro-inflammatory cytokines and development of cytotoxic T lymphocytes against host allo-antigens [41–43]. During 14-day subcutaneous treatment with ATL146e as a GVHD preventative treatment, weight loss and overall mortality was reduced in agonist treated mice over vehicle treated controls. In addition, visible signs of GVHD, which include hunched posture, diarrhea and skin inflammation, were all reduced. At the molecular level, alloreactive donor T cell activation was also inhibited by ATL146e treatment. Pro-inflammatory cytokine levels in blood serum were significantly reduced below vehicle treated mice, with an accompanying increase in the anti-inflammatory cytokine IL-10. Treatment of a GVHD model with ATL146e 9 days after HSCT reversed weight loss, indicating that ATL146e can also serve as a treatment for established GVHD. This could be a very promising new treatment for patients who suffer from steroid refractory GVHD, which is also usually unresponsive to other more common GVHD treatments.

Further studies have helped to elucidate the relationship between treatment with A_2A_R agonists and the immunosuppressive response. The treatment of naïve CD4^+^CD25^−^ mouse T cells in vitro with the A_2A_R agonists ATL146e, ATL370 and ATL1223 have been shown to enhance the TGFβ-induced generation of FoxP3^+^ T_regs_ [2]. When a GVHD mouse model was similarly treated, there was significant improvement in weight and mortality as well as inhibition of pro-inflammatory cytokine and chemokine production. Most interesting was the increase in FoxP3^+^ T_regs_ in agonist treated mice when compared with vehicle controls. It was found that T_regs_ increased in agonist treated mice were of donor origin, confirming the requirement for the development of functional T_regs_ from donor cells to prevent increased mortality in GVHD. This increase of T_regs_ was observed not only in lymphoid tissues and blood, but also in two major target organs of GVHD, the skin and colon, which both showed a marked improvement over vehicle treated controls.

It was also observed that the pro-inflammatory cytokines GCSF and IL-6 were reduced in serum after treatment, and that the tolerance-inducing cytokine IL-10 was elevated. Possibly most important was the observation that IFNγ production after restimulation is preserved with ATL146e treatment, which could explain the lack of susceptibility of these mice to infection even after long-term treatment with the immunosuppressive A_2A_ receptor agonist [3]. Since it has been observed that long-term activation of A_2A_ receptors on CD4^+^ T cells by cAMP elevating agents results in sustained tolerance in CD4^+^ T cells without impairing the immune response to non-specific mitogens, it is likely that activation of the A_2A_ receptor preserves T cell response to global stimuli while preventing T-cell response to specific alloantigens. This is an important observation, as it confirms that long-term administration of A_2A_R agonists does not cause inappropriate termination of immune responses despite the importance of A_2A_R activation in the anti-inflammatory negative feedback loop.

Study of the A_2A_R pathway has determined that the activation of A_2A_R results in a reduction of single positive CD4 or CD8 T_eff_ cells, which are responsible for the release of many pro-inflammatory cytokines, and that T cells activated in the presence of A_2A_R agonists are unable to proliferate or produce IL-2 or IFNγ upon restimulation [3]. These same T_eff_ cells also show reduced proliferation in the presence of A_2A_R agonists, reducing autoimmune responses from these cells [29]. In addition, A_2A_R agonists tend to produce T_regs_ that are responsible for induction of immune tolerance by reducing the production of IL-6 and increasing the production of TGFβ, which also serves to further inhibit the generation of adaptor T effector cells [2]. These recent studies have shown that activation of the A_2A_ receptor and downstream events seems to be composed of two branches: (1) inhibition of donor T cell activation and migration to target organs and (2) induction of anti-inflammatory FoxP3^+^ regulatory T cells.

## 6 GVHD and A_2A_R agonists: mechanisms of function

**Fig. 1:**
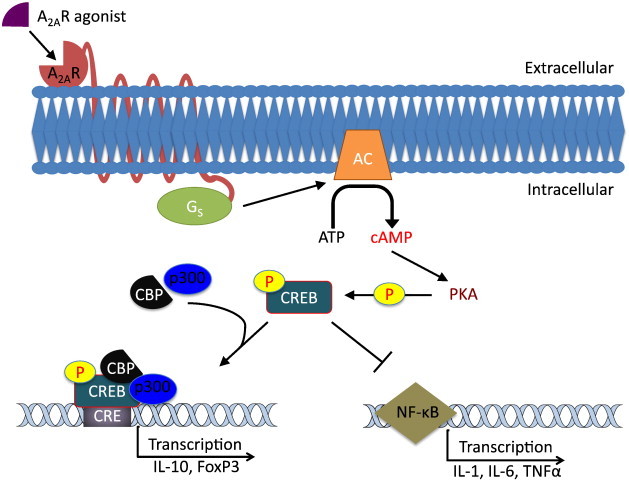
A proposed mechanism for the adenosine A_2A_R agonist mediated activation of the cAMP–CREB pathway and induction of immune tolerance. The agonist binds to the A_2A_ receptor and activates the Gs protein. The activated Gs protein interacts with adenylyl cyclase (AC), which converts ATP to cAMP. Elevation of intracellular cAMP levels activates protein kinase A (PKA), which phosphorylates the cAMP response element binding protein (CREB) at Ser-133. The phosphorylated CREB expresses a dual function in promoting tolerance. First, by forming a complex with p300 and the nuclear co-factor CBP, CREB is able to bind to CREB response elements (CREs) and activate transcription of anti-inflammatory cytokines such as IL-10 as well as increasing the expression and number of FoxP3^+^ T_regs_. Secondly, activated CREB blocks the NF-κB pathway, which is responsible for the transcription of pro-inflammatory cytokines such as IL-1, IL-6 and TNFα.

The mechanism behind the activity of A_2A_R agonists in GVHD has not yet fully been elucidated, but much is known about the activity of the receptor in other diseases. From studies performed using adenosine and adenosine analogs, a mechanism can be proposed for the function of A_2A_R agonists in GVHD. The A_2A_ receptor, which is coupled to a Gs protein, is activated by the binding of the agonist, causing a significant increase in intracellular cAMP levels generated by adenylyl cyclase (AC) during the inflammatory response (Fig. 1). This elevation of cAMP activates protein kinase A (PKA), which results in the phosphorylation and activation of the cAMP response element-binding protein (CREB) at Ser-133 [44]. CREB creates a complex with p300 and the nuclear co-factor CBP, which in turn binds to cAMP responsive elements in the promoter region of many genes, including the anti-inflammatory cytokine IL-10. CREB has also been shown to promote TGFβ mediated generation of FoxP3 T_regs_ [45–48]. Normally, the T_reg_-specific demethylated region (TSDR) in the FoxP3 locus remains methylated in resting T cells, preventing CREB from binding. TGFβ signaling removes this methylation, allowing CREB to bind and transcribe FoxP3, promoting the development and stabilization of T_regs_.

In addition, CREB is able to indirectly modulate cytokine gene expression through the inhibition of nuclear factor-κB (NF-κB) [49]. Blocking of the NF-κB pathway prevents the expression of many pro-inflammatory cytokines including TNFα. Though the precise mechanism is unclear, it is very likely that the inhibition of the NF-κB pathway in conjunction with CREB-induced T_reg_ production is the primary method by which A_2A_ receptor agonists prevent the release of tissue damaging cytokines and reduce the severity of GVHD in murine models.

## 7 The future of A_2A_R agonists in GVHD treatment

Currently, the mainstay of treatment for GVHD consists of corticosteroids, often in conjunction with other immunosuppressants. While this is effective in reducing the donor T cell reaction to host tissues, it is only effective in 50% of patients and opens up the immune system for secondary infection. There are also significant side effects associated with administration of these drugs. Data suggests that the use of A_2A_R agonists in the clinic could increase donor immunotolerance to host tissues while maintaining an immune response to foreign pathogens. The majority of deaths in GVHD patients are a result of unrelated infections that form as a result of suppressed immune systems as well as due to the immune dysregulation caused by the GVHD itself. Implementation of A_2A_R agonists in the clinic either in conjunction with corticosteroids, or hopefully as a steroid sparing agent, may provide a novel prevention or treatment strategy that both prevents or treats GVHD and reduces the risk of infection.

To date, much of the data collected on A_2A_R agonists indicates that these agents are promising as a therapeutic treatment for GVHD. Despite this, there are still questions to be answered, and determining the exact mechanism by which this immune tolerance is activated will be important to improving the quality and potency of future agents. Two obstacles in particular stand in the way of pharmacologic progress with A_2A_R agonists: (1) A_2A_R agonists tend to have very short half-lives, which results in the need for additional treatments to replenish active drug levels and (2) A_2A_R are abundant on many cell types, including those found in the brain and heart, and the specificity is a concern for A_2A_R agonists to prevent neuro- or cardiotoxicity. These concerns have recently been addressed in a study involving the attachment of a small Fc domain protein to the A_2A_R agonist, conferring stability and preventing non-specific binding of the agent [50]. In this study, the Fc bound agonist was able to successfully treat a murine model of pneumonitis to an equivalent efficiency as that of an unbound agonist. In addition, only low levels of Fc bound agonist were detectable in the heart and brain of treated mice, showing high specificity of the Fc domain for areas of inflammation. This method of conjugating A_2A_R agonists to the Fc domain is another step towards creating agents for the treatment and/or prevention of GVHD in human patients and may also serve as a general method to target delivery of small molecule drugs to areas of inflammation.

A_2A_ receptor agonists, through activation of T_regs_ and immune tolerance, have proven to be effective in reducing and preventing GVHD development in murine models of the disease. These results suggest that A_2A_R agonists have therapeutic potential for the treatment of acute GVHD in human patients and possibly other autoimmune disorders. Currently, humanized mouse models are being developed to further elucidate the effects of A_2A_R agonists in a model more reflective of the human immune system. These models will also help us to determine the effects of long term administration of adenosine receptor activation by A_2A_R agonists in human immune cells. These new models, along with the development of more human specific compounds, will lead us forward in creating clinical agents for use in phase 1 and phase 2 patient studies.
